# The Emerging Role of Virtual Reality as an Adjunct to Procedural Sedation and Anesthesia: A Narrative Review

**DOI:** 10.3390/jcm12030843

**Published:** 2023-01-20

**Authors:** Rita Hitching, Hunter G. Hoffman, Azucena Garcia-Palacios, Maheen M. Adamson, Esmeralda Madrigal, Wadee Alhalabi, Ahad Alhudali, Mariana Sampaio, Barry Peterson, Miles R. Fontenot, Keira P. Mason

**Affiliations:** 1 School of Medicine and Public Health, University of Newcastle, Newcastle, NSW 2308, Australia; 2 Department of Mechanical Engineering HPL, University of Washington, Seattle, WA 98195, USA; 3 Department of Computer Science, Faculty of Computing and Information Technology, King Abdulaziz University, Jeddah 21589, Saudi Arabia; 4 Department of Basic Psychology, Clinic and Psychobiology, Jaume I University, 12071 Castellon de la Plana, Spain; 5 WRIISC-WOMEN and Rehabilitation Service, Veterans Affairs Palo Alto Health Care System, Palo Alto, CA 94304, USA; 6 Department of Neurosurgery, Stanford University School of Medicine, Stanford, CA 94305, USA; 7 Rehabilitation Service, Veterans Affairs Palo Alto Health Care System, Palo Alto, CA 94304, USA; 8 Immersive Virtual Reality Research Group, King Abdulaziz University, Jeddah 21589, Saudi Arabia; 9 Department of Computer Science, School of Engineering, Computing and Informatics, Dar Al-Hekma University, Jeddah 21589, Saudi Arabia; 10 Department of Psychology, University of Coimbra, 3000-115 Coimbra, Portugal; 11 Department of Social Work, Catholic University of Portugal, 1649-023 Lisbon, Portugal; 12 Department of Veterans Affairs, Reno, NV 89502, USA; 13 Department of Anesthesiology and Pain Medicine, University of Washington, Seattle, WA 98195, USA; 14 Department of Anesthesiology, Critical Care and Pain Medicine, Harvard Medical School, Boston Children’s Hospital, Boston, MA 02115, USA

**Keywords:** non-pharmacologic, analgesia, sedation, anesthesia, virtual reality

## Abstract

Over the past 20 years, there has been a significant reduction in the incidence of adverse events associated with sedation outside of the operating room. Non-pharmacologic techniques are increasingly being used as peri-operative adjuncts to facilitate and promote anxiolysis, analgesia and sedation, and to reduce adverse events. This narrative review will briefly explore the emerging role of immersive reality in the peri-procedural care of surgical patients. Immersive virtual reality (VR) is intended to distract patients with the illusion of “being present” inside the computer-generated world, drawing attention away from their anxiety, pain, and discomfort. VR has been described for a variety of procedures that include colonoscopies, venipuncture, dental procedures, and burn wound care. As VR technology develops and the production costs decrease, the role and application of VR in clinical practice will expand. It is important for medical professionals to understand that VR is now available for prime-time use and to be aware of the growing body in the literature that supports VR.

## 1. Introduction

In the past 20 years, sedation delivery outside of the operating room has evolved in a number of ways designed to reduce sedative side effects [1]. Risks of significant adverse events have been reduced by a number of improvements in safety, such as the better identification of which patients can safely be treated outside of the operating room, training, physiologic monitoring, and broadened sedative options [2,3,4,5,6,7,8,9]. For example, the use of intranasal dexmedetomidine for the sedation of children during nonpainful imaging procedures [10] and the use of adjunctive dexmedetomidine during painful procedures, e.g., to reduce reliance on opioids [11,12,13,14,15,16].

The evolution of national guidelines, standardized definitions of the depth of sedation and outcomes, and the application of non-pharmacologic techniques have further contributed to reducing the risk of significant adverse events [17]. Once common practices such as immobilization through papoosing (physically restraining a child to keep children still during the medical procedure) have almost universally been replaced with distraction techniques that range from the rudimentary (a book) to the more sophisticated (tablet) [18].

The development of more powerful non-drug adjuncts has intensified, in light of the following Food and Drug Administration (FDA) warning [19]. “The FDA is warning that repeated or lengthy use of general anesthetic and sedation drugs during surgeries or procedures in children younger than 3 years or in pregnant women during their third trimester may affect the development of children’s brains” (FDA, 2017 p 1); see also [20]. In children, the untoward effects of general anesthesia and procedural sedation have been reported to include short-term behavioral and emotional changes, a decline in academic achievement, maladaptive behavior (eating and sleeping difficulties, withdrawal, apathy, enuresis), and fear of future medical procedures [21,22,23,24,25,26,27].

With the heightened media coverage, fueled by the above-mentioned FDA warning concerning the possible deleterious effects of anesthesia and sedation on the developing neonate and infant brain, there is an increased urgency to consider new approaches to decrease anesthetic exposure.

There is growing evidence that the patient’s psychological state of mind can influence the dosage of sedatives needed to achieve the target sedation level. For example, a patient having a mild panic attack as they enter the operating room is likely to be more challenging to sedate than a patient who is calm and collected as they are being sedated. Over the past decade, help from child life specialists and other non-pharmacologic techniques have proliferated in their presence in the pre-procedural and pre-operative areas [28,29]. Clowns have become increasingly visible in these areas, entertaining the children to relieve parental and patient anxiety, with the goal of reducing pharmacologic analgesics and anxiolytics [30,31]. Many traditional distraction techniques are modestly helpful, but a more powerful distraction with little or no side effects is greatly needed. As briefly reviewed in the current narrative review paper, immersive virtual reality appears to be an unusually effective technique and is quickly becoming a “distraction on steroids”. According to Websters Dictionary, Virtual Reality (VR) is “an artificial environment which is experienced through sensory stimuli (such as sights and sounds) provided by a computer and in which one’s actions partially determine what happens in the environment.” [32]. There is currently intense interest in the application of immersive virtual reality as a non-pharmacologic technique to reduce the need for anxiolytic, analgesia, and sedation delivery in the pre-procedure/operative period and to help reduce post-operative pain and reduce reliance on medications. Immersive VR is increasingly being used as a non-drug analgesic, anxiolytic, and digital sedative for procedural sedation. With the application of VR, patients have reported reductions in pain and anxiety during painful medical procedures [33,34,35] and VR may help reduce reliance on opioids for pain management [36,37]. The application of VR for the pediatric population, as an adjunct or in some cases as a replacement to procedural sedation, could offer important benefits [38].

This narrative review briefly explores some of the relevant literature on VR applications and discusses how the recent increase in usability and affordability is facilitating the proliferation of VR in peri-operative and surgical environments. The current brief narrative review is an overview and synthesis based on information from a selection of individual published scientific studies, meta-analyses, and personal experience of the originator of VR analgesia. This review presents a broad perspective on the topic of virtual reality analgesia for acute pain control, especially during procedures that often involve sedation. This paper also includes a brief history of several large steps of development of using VR pain management over the past 20 years. This review targets clinicians and is designed to help bring practitioners of pediatric sedation up to date on growing opportunities to use VR in their clinical practice.

## 2. Virtual Therapeutic

**Figure 1:**
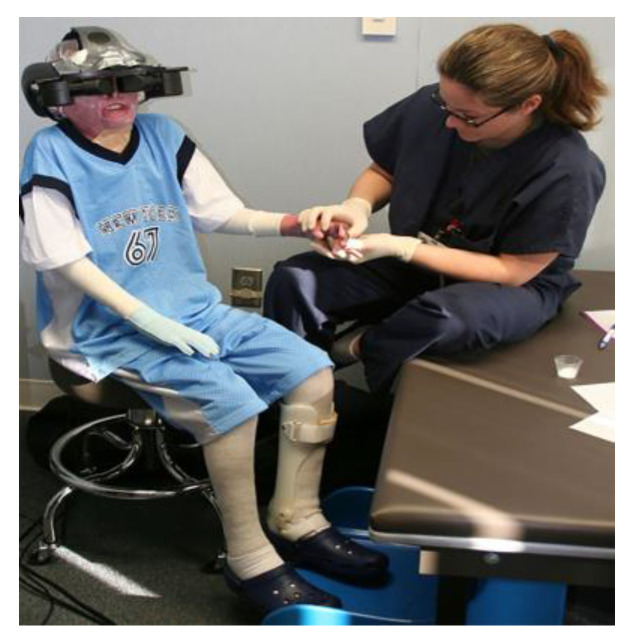
A pediatric burn patient using VR analgesia during a painful medical procedure. Photo and copyright Hunter Hoffman, www.vrpain.com accessed on 28 November 2022.

**Figure 2:**
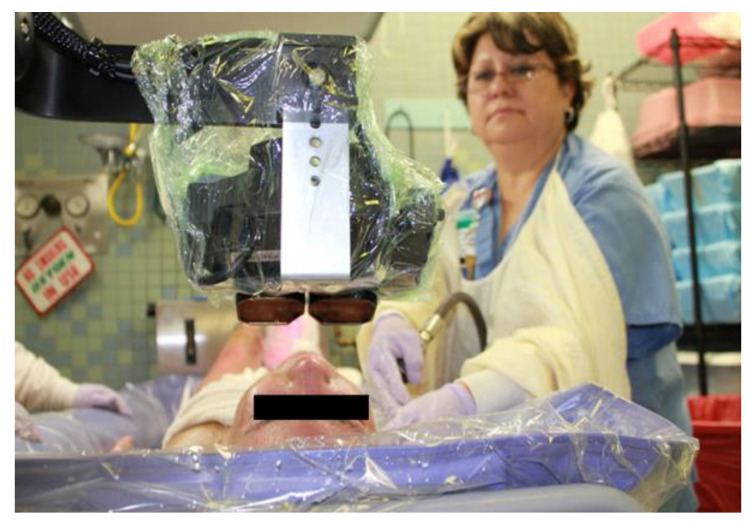
Some VR systems have been customized to be used during wound care during wound debridement. Some patients have face or head burns that cause it to be difficult to wear traditional head-mounted VR goggles. The “articulated arm” shown above holds the goggles near the patient’s eyes, with little or no contact with the patient [35]. Photo and copyright Hunter Hoffman, www.vrpain.com, accessed on 28 November 2022.

The application of VR as a non-pharmacologic anxiolytic and peri-analgesic technique, has evolved over the past decade. At its essence, patients don VR goggles (see Figure 1) and interact with virtual objects in a computer-generated world. During VR physical therapy (where motion is desired), with hand, head, and body movements, patients are able to transform their environment: stir the witches’ cauldron, dance with a robot, walk a pirate’s plank and pick up treasures, etc. However, with regard to sedation, VR experiences have been customized to minimize physical body movements. The patients use eye-movements and/or mouse tracking, e.g., Hoffman et al., 2019 [35] to aim snowballs at virtual snowmen and click a mouse button to throw snowballs, see Figure 2. Several VR systems have been tailored to the procedure, specifically designed to distract patients from the painful or anxiety generating stimulus without interfering with the wound care nurse’s ability to work on the patient. For example, eye tracking can help patients interact with the virtual world, while the patient remains physically still during the medical procedure [39].

**Figure 3:**
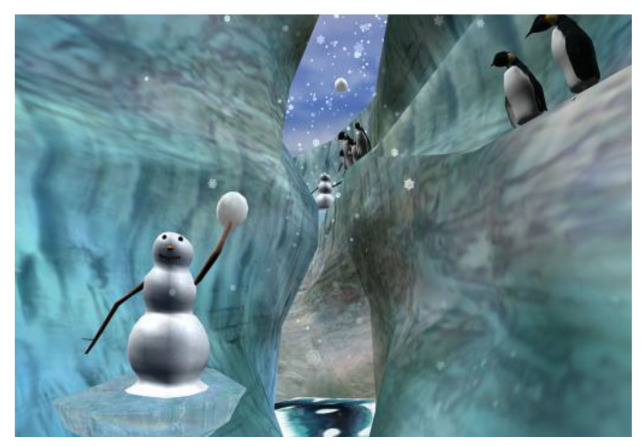
The most recent version of SnowWorld, image by Ari Hollander and Howard Rose, copyright Hunter Hoffman, www.vrpain.com, accessed on 28 November 2022. SnowWorld is the first immersive virtual reality world specifically designed for pain reduction. www.vrpain.com, accessed on 28 November 2022.

To track eye movements, six small infrared lights embedded inside the VR goggles are shined onto the surface of the patient’s eyes, creating a pattern of dots on the outer eye. The pattern changes as they look in different directions. The changes in where the patient is looking are then captured using miniature video cameras pointed at the patients’ eyes [39]. The computer chip in the VR goggles can tell from the patterns what virtual objects the patient is looking at in VR, such as to aim virtual snowballs. VR is designed to transport the patient to another reality, immerse them in their new virtual environment, and distract them by eliciting their engagement in an interactive game designed around their new environment. VR transports the patient to another 3-dimensional reality, e.g., SnowWorld [35,40], Mindfulness River World [41,42], or VR Animal Rescue World [43]. Even the slightest eye movement or mouse movement can change where the patients are looking in VR and allow patients to interact with objects in VR. Interactivity increases people’s illusion of “being there” in the computer-generated world, as if it is a place they are visiting [44]. The patients describe a unique sense of “being present” in this computer-generated world. VR transports patients from their unpleasant, anxiety, and pain-provoking environment to an alternate, engaging, and entertaining computer-generated virtual world designed to help patients think about something other than their pain. VR can reduce acute pain and anxiety related to the most painful procedures: the scrubbing, cleaning, and debridement of burn wounds [35,45] or venipuncture [46,47]. Burn wound care is typically severely painful, despite the use of powerful opioid analgesics. Snow World VR is a frigid polar climate within which patients throw snowballs at targets (e.g., snowmen, penguins, and wooly mammoths, see Figure 3). SnowWorld is tailor-made to distract patients from the flashbacks of fire and the pain associated with the dressing change. VR analgesia enables patients to tolerate these procedures without increasing traditional analgesics.

The literature on the safety, efficacy, and acceptability of VR has grown significantly in recent years [48,49]. For example, in children presenting for surgical procedures, VR has been shown to alleviate preoperative anxiety and facilitate the induction of anesthesia [50,51]. A review of outcomes in 213 children (6–18 years), primarily in the perioperative (60%, n = 128) and clinic (15%, n = 32) settings, demonstrated that VR-related adverse events were rare, self-limiting, and minor, such as occasional increases in anxiety (3.8%, n = 8), nausea (0.5%, n = 1), and dizziness (0.5%, n = 1) [25].

Importantly, the costs of developing, creating, and implementing VR has decreased greatly over the past decade, thereby increasing its applicability and affordability as a cost-effective tool for clinical practice [49,52]. The increased feasibility of widespread dissemination is occurring at a time of greatly increased demand for non-pharmacologic analgesics, in light of the opioid death epidemic [53,54] and a growing awareness of the need to avoid oversedation.

The mechanism of action of immersive VR is not yet fully understood, but is thought to be related to VR’s ability to divert attention [34,44]. fMRI studies demonstrate that VR significantly reduces pain-related brain activity in the anterior cingulate cortex, primary and secondary somatosensory cortex, thalamus, and insula [55,56,57] and show that VR can provide equi-analgesia to hydromorphone.

## 3. VR as a Non-Pharmacologic Anxiolytic, Analgesic, Sedative

VR reduces procedural anxiety [58] by redirecting the patient’s attention into the computer-generated world, as the VR “transports” the patient from the clinical setting to another ‘reality’ [44]. The application of VR has been shown to decrease diastolic blood pressure, heart and respiratory rate, temperature, muscular tension, temperature, skin conductance, and serum carbon dioxide levels [59]. VR has been used during a wide range of medical procedures.

The current non-exhaustive narrative review briefly explores some VR studies using VR during several medical procedures commonly using sedation outside of the operating room: with a focus on colonoscopies, venipuncture, dental procedures, MRI scans with autistic children, and wound care of children hospitalized with severe burn injuries.

## 4. VR Sedation during Colonoscopies

In the United States, it is estimated that 15 million colonoscopies are performed annually; it is a recommended medical procedure to screen for the third most commonly diagnosed cancer [60]. However, many people who could benefit from the preventative removal of pre-cancerous polyps avoid receiving their colonoscopy, for fear it will be unpleasant [61]. Some of these people who avoid colonoscopies die of colon cancer that could easily have been prevented via endoscopic polyp removal. In one recent survey about why patients avoid receiving a colonoscopy “*Anxiety was as a key barrier cited by patients and SSPs, arising from the moment the patient received the invitation letter. Notably, procedural-related anxieties centred upon the fear of pain and discomfort and test invasiveness.*” ([62] p. 1).

Colonoscopies are most frequently prescribed to older adults. Additionally, there is in fact some cause for concern about rare but real side effects of sedation often used during colonoscopies. The moderate and deep sedation, as well as general anesthesia, that are utilized to achieve these procedures all carry inherent associated-risk [63,64,65]. A systematic review of four randomized clinical trials (RCT) found that VR was superior to the controls and had similar efficacy to traditional pharmacologic sedation [66].

## 5. VR Distraction during Venipuncture

For the induction and maintenance of anesthesia and sedation, an intravenous (IV) catheter is often placed in the pre-operative or pre-procedure location (e.g., in the patient’s forearm). Needle phobia is common amongst toddlers, children, and some adolescents. Venipunctures can elicit exaggerated anxiety, and the fear of needles has contributed to the avoidance of healthcare and reduced adherence to childhood immunization schedules [46,47,67,68]; an unpleasant venipuncture can set a sedation off to an unpleasant start. Immersive VR can alleviate pain and anxiety in children and reduce the parents and nursing staff ratings of the childrens’ procedural pain and anxiety [69]. In a randomized controlled trial comparing the feasibility and efficacy of incorporating VR into the routine care of venipunctures in 143 children (10–21 years old), VR significantly reduced acute procedural pain and anxiety. VR reduced caregiver and patient distress, increased clinician satisfaction, and improved efficiency and throughput in the outpatient phlebotomy clinics. These results are consistent with other recent applications of VR for venipuncture [46,68,70,71,72]. Similarly, children undergoing blood draws and intravenous placement in the emergency department reported significantly greater reductions in fear of pain using immersive VR than the active control conditions—watching television or receiving standard distraction. Although children reported significantly less fear and greater satisfaction during VR compared to active control, no significant reductions in pain intensity were noted in that study [73]. In another study, VR did not reduce pain in children undergoing procedural sedation under local anesthesia [38].

## 6. VR Sedation during Dental Procedures

VR has been used with success in the dental setting for children and adult patients presenting with mild to moderate fear and anxiety: VR distraction reduced the self-reported levels and physiological indicators of anxiety, fear, and pain [47,59,74]. In the pediatric population, VR decreased reported pain and anxiety levels with accompanying decreases in the pulse rate and oxygen saturation before, during, and after restorative dental treatment (*p* < 0.0001) [75]. Similar responses were reported in healthy children requiring buccal infiltration anesthesia [76] and inferior alveolar nerve block (IANB) for mandibular tooth extraction [77]. In another study of the analgesic effect of VR, the patients undergoing periodontal scaling and root planing procedures reported significantly reduced pain perception compared to an active comparator—watching a movie—group and passive controls (*p* < 0.001) with accompanying reductions in blood pressure and pulse rate (*p* < 0.001) [78]. In another study, children undergoing dental extractions did not report a significant reduction in anxiety [79].

## 7. VR Sedation Is Especially Challenging in Children with Autism

VR has been used with success for children with autism spectrum disorders (ASD). Children with ASD can display delayed or repetitive language, sensory sensitivity, and elevated anxiety levels [80,81,82]. Repetitive behaviors such as rocking, jumping, twirling, pacing around, and other “hyper” and impulsive, sometimes aggressive behaviors cause it to be challenging for children with autism to stay physically still during medical procedures (e.g., MRI scans). VR distraction has been used in this clinical setting for dental procedures of children with ASD, alleviating the accompanying behavioral challenges during the procedures [83]. The cognitive and behavioral challenges associated with ASD can limit the child’s ability to tolerate other medical procedures often necessary to evaluate their comorbidities (immune, gastrointestinal, and neurologic disorders) [84,85]. Magnetic Resonance Imaging (MRI) studies can be challenging for patients with autism, who often require immobility for extended periods of time in the cold, noisy, and claustrophobic MRI environment. With the FDA and Society for Pediatric Anesthesia warnings on the potential for neurotoxicity of sedation drugs to the developing brain, parents can be reluctant to consent to traditional pharmacologic anesthesia or sedation [86,87,88,89]. If unsedated autistic patients move around during imaging, the result can be aborted or produce blurry scans and the reduced quality of medical care.

Days prior to their MRI scans, training in simulations can help familiarize autistic children with the MRI environment and can help them remain more calm during subsequent scans. For example, autistic children can practice being in an fMRI at home [89]. VR distraction and other types of distraction may also help enable children with ASD to successfully complete an MRI (see related pilot study of successful use of distraction and VR with autistic children [90], and successful SnowWorld VR distraction of clinically claustrophobic adults during mock MRI scans [91].

## 8. Transnasal Endoscopies and PICC Line Insertions

In addition to radiological imaging studies, VR has been used as an adjunct to pediatric gastrointestinal procedures. Upper esophagogastroendoscopies are frequently performed with sedation or general anesthesia in the pediatric population [92]. Children with eosinophilic esophagitis typically require frequent follow-up endoscopies to follow disease progression. In these patients, some studies have demonstrated that VR has eliminated the need for sedation or anesthesia and for transnasal endoscopy and reduced the associated costs [93]. Transnasal endoscopy involves inserting a tube into the patient’s body through their nose.

Peripherally Inserted Central Catheter (PICC) lines are thin flexible tubes inserted peripherally (e.g, into the arm) until one end is near the larger veins near the patient’s heart (e.g., to inject intravenous fluids, to give blood transfusions, chemotherapy, and other drugs, and to take blood samples). In a group of 10 children, when VR distraction was used, a Peripherally Inserted Central Catheter line insertion did not require patient restraint and VR was associated with reductions in anxiety and greater caregiver satisfaction [94].

## 9. Analgesic Potential of VR during other Painful Medical Procedures

VR is increasingly being used as an adjunct or alternative to pharmacologic analgesia and sedation for a wide range of medical procedures [95]. VR distraction has been incorporated into a multimodal approach to the management of procedural pain: distracting the patient’s focus to spend less time thinking about pain, while increasing their pain threshold and tolerance, and causing medical procedures to be significantly more fun [45]. The patients continue to benefit from VR when it is used repeatedly [35]. VR has been used as an anxiolytic and analgesic adjunct for orthopedic outpatient surgical procedures [36] as well as for transurethral microwave thermotherapy in elderly patients [96].

In adults, VR has been successfully used as an adjunct to procedural sedation for lumbar punctures, transcatheter aortic valve implantation (TAVI), and uterovaginal brachytherapy [95,97,98]. Adults undergoing regional nerve blocks for upper extremity surgery prefer VR, with higher post-operative satisfaction scores (*p* < 0.001), lower anxiety scores (*p* < 0.001), and more stable hemodynamics (*p* = 0.031) [99].

A study investigating the feasibility and effectiveness of VR for pain associated with atrial fibrillation (AF) ablation under conscious sedation in a group of 48 patients (mean age 63.0, SD 10.9 years; n = 16, 33.3% females) reported lower mean perceived pain (3.5 [SD 1.5] vs. 4.3 [SD 1.6]; *p* = 0.004) and greater comfort (7.5 [SD 1.6] vs. 6.8 [SD 1.7]; *p* = 0.03) than control. Although VR significantly reduced pain and was not associated with procedural complications or an increase in fluoroscopy duration, VR was not associated with any reductions in morphine consumption [100].

By reducing or in some cases eliminating the need for sedation, VR can lower the incidence of apnea [101]. In addition, studies of adults undergoing knee replacement have demonstrated improved intra-operative hemodynamics (reduced hypotension), decreased fentanyl requirement, and lower post-operative pain scores [102,103].

## 10. VR Sedation/Analgesia during Burn Wound Cleaning

VR has been trialed for its potential to provide anxiolysis, analgesia, and distraction for severe burn patients. In the pediatric population, burn wound care is a profoundly painful and traumatic experience [35,104]. Burns are a leading cause of emergency department visits and hospitalizations (e.g., scalds and contact with hot household appliances) [105]. Traditional pharmacologic analgesics (opioid and non-opioid) and adjuncts (e.g., nonsteroidal anti-inflammatory drugs, benzodiazepines, neuroleptics, and opioids) are often unable to control the pain associated with wound care and debridements [106]. Although nearly all of the research on VR analgesia for pediatric burn patients has studied children of 6 years and older, a hybrid projector-based VR protocol and desktop VR have even demonstrated a significant effect in reducing pain related to hydrotherapy procedures in very young pediatric burn injuries as young as 2 years old [43,107].

## 11. VR for Military and Veteran Patients

The military has long used VR for training. The U.S. Army recently purchased 22 billion dollars of Microsoft Hololens goggles, which can be used for either VR or see-through displays that superimpose computer-generated virtual images onto the users view of the real world, which is known as Augmented Reality (AR) [108]. There is also growing interest from the U.S. Office of Veterans Affair in using VR to help reduce the acute and chronic pain of U.S. veterans [109,110]. The military is very interested in acute pain management techniques that do not cloud the soldiers decision process and that facilitate deployment readiness [111,112], see also [109]. The Tactical Combat Casualty Care Guidelines are the “standard of care for the modern battlefield” [113]. Peterson et al., 2021, p. 11 [109] recommend revising the current Tactical Combat Casualty Care Guidelines to include “the addition of VR as an effective and hemodynamically safe approach to the current management of acute trauma pain in military personnel during medical procedures”.

Additional research and development of VR analgesia designed to meet the unique needs of military and VA patients, including both acute and chronic pain, is recommended [114,115], see also [116].

## 12. Meta-Analyses of VR Distraction

Systematic reviews and meta-analyses demonstrated that immersive VR provides superior analgesia to controls during dressing changes/wound care in hospitalized children and adolescents (Cohen’s d = −0.94, a large effect size (95% CI = 0.62, 1.27; Z = 5.70; *p* < 0.00001) compared to the control. Fully immersive VR is considered to be a useful adjuvant in pediatric burn care [117]. These recommendations are supported by another meta-analysis of 18 studies that demonstrates VR efficacy in reducing procedural pain associated with burn care (Cohen’s d = −0.49; a medium effects size, 95% CI −0.78, −0.15; *I*^2^ = 41%), and VR analgesia of procedural pain during burn care [118].

A systematic review and meta-analysis of 16 studies reported on the efficacy of VR for alleviating pain and anxiety in children undergoing a range of medical procedures (venous access, burn, dental, and oncology care). The results showed large effect sizes in favor of VR at reducing children’s self-reported pain (Cohen’s d = 1.30, a large effect size (95% CI, 0.68–1.91) and anxiety (Cohen’s d = 1.32, a large effect size (95% CI, 0.21–2.44). The results are consistent with caregivers’ (Cohen’s d = 2.08, a large effect size; 95% CI, 0.55–3.61) and professionals’ (Cohen’s d = 3.02, a large effect size; 95% CI, 0.79–2.25) observations of the children’s pain levels [119]. However, note that these unusually large effect sizes are due in part to biases frequent in these relatively early studies in this research program.

The clinical efficacy of VR in managing pediatric procedural anxiety and pain is further supported by a recent literature review primarily for burn wound care and post-burn physiotherapy, needle-related, and dental procedures between 2000 and 2020. This review supported the efficacy of VR in addressing procedural pain and anxiety in children aged from 6 months to 18 years. The authors concluded that VR is redefining pain and anxiety management with nurses, who may play a leading role in the implementation of VR into clinical care [120].

VR improves the perioperative patient experience and shows promise at reducing intra-operative anesthetic requirements. A systematic review and meta-analysis of 20 experimental and quasi-experimental trials published between January 2007 and December 2018 on VR as an analgesic agent in acute and chronic pain in adults showed benefits of VR to reduce peri- and postprocedural acute pain [121]. Across all trials included in the meta-analyses, despite a high degree of statistical heterogeneity, VR was associated with reductions in pain scores, with a moderate effect size (Cohen’s d = −0.49 (95% CI −0.83 to −0.41, *p* = 0.006) [114].

Until recently, technological limitations of VR have been a barrier to dissemination. Due in part to the ongoing opioid overdose death crisis [122], VR analgesia/sedation is currently a research topic of intense scientific and clinical investigation. Over the past 25 years, despite growing empirical evidence of its efficacy, VR has not been widely adopted in everyday clinical practice. This is partly because, until recently, the VR systems were very rare, expensive, and complicated. During the early days of VR [33,34], the VR system was heavy and costly (USD 60,000 for one VR system), and included a heavy computer monitor, a 55 lb Silicon Graphics supercomputer, an 8-pound VR helmet, and a separate USD 8000 Polhemus FastTrak electromagnetic tracking system. The transport and set-up required several hours and was labor intensive and required considerable technical trouble-shooting skills. Over the years, VR technology has evolved and improved, becoming much lighter weight (less than 2 pounds total for the entire VR system), less expensive, and requiring less technological sophistication to set up, but, until recently, VR was still not easy for nurses to use without help, was still very expensive for the wide field of view VR helmets, and most patients who received VR were participants in research studies.

**Figure 4:**
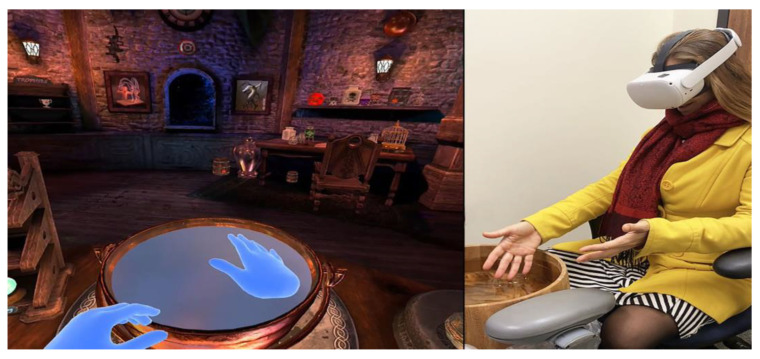
Above right is a photo of the Meta Quest2 VR helmet. This lightweight helmet weighs only 17.7 ounces, is wireless, and uses cameras to optically track head and hand movements. Image on left shows a screenshot from an optically hand-tracked game named Waltz of the Wizard, aldin.com, accessed on 28 November 2022, photo on right by Hunter Hoffman, both images copyright Hunter Hoffman, www.vrpain.com, accessed on 28 November 2022.

Some major breakthroughs in VR technology occurred in 2016, as high-tech companies began mass producing VR helmets and mass marketing. In 2019, an untethered VR system was released that was wireless, self-contained, and easy to use for novice computer users (e.g., nurses). The Oculus Quest (2019) and Oculus Quest2 (2020) VR systems (see Figure 4) are now very inexpensive and widely available worldwide and are increasingly being used for VR analgesia. So, the biggest technological limitations to the dissemination of VR pain and anxiety reduction have recently been overcome and are continuing to improve (e.g., miniaturization and increased resolution, camera-based hand tracking, face tracking, eye tracking, improved graphics, etc). The stand-alone Meta Quest2 now costs under USD 500 per helmet and does not require a laptop. The new VR systems with eye tracking and hand tracking are more immersive and more distracting [39,44] and the immersiveness of VR (and the analgesia effectiveness) is currently increasing dramatically each year, due in part to multibillion dollar investments and competition amongst the big tech companies. For example, one single company, Facebook, has invested well over USD 2 billion into VR technology so far and it has announced that, as of July 2022, they have sold over 15 million Quest2 VR helmets Worldwide in less than 2 years. Although Facebook stock has recently tumbled in 2022, several other well-known tech companies such as Apple, Sony, Microsoft, Google, and Samsung are also creating VR technologies anticipating potentially lucrative new VR-related markets [123].

## 13. Conclusions

As a result of huge private investments into the hardware by high tech companies such as those just mentioned and custom software by researchers and small VR therapy companies customized to benefit patients, VR has become an affordable tool that could substantially improve patient outcomes. Future clinical research and the development of VR is critical as we seek means to improve the patient care experience and minimize their risks and discomfort. As briefly reviewed in the current narrative study, immersive VR can help reduce anxiety before medical procedures, can help reduce anxiety and pain during medical procedures, and can help reduce post-surgical pain. Between 1998 and 2016, a growing body in the literature of clinical studies, including Mayday Fund and NIH-funded research, has shown large reductions in pain and anxiety during painful medical procedures. However, until 2016, VR was rarely used in clinical practice and most patients able to use VR were research subjects. Many of the most serious barriers that prevented more widespread use of VR in everyday clinical practice (e.g., very high expense, difficult to use, and bulkiness) have recently been eliminated. Because of multibillion dollar investments by private industries, VR technology is currently improving at a fast pace, thus increasing its potential for medical dissemination. Easy to use, highly distracting, wireless, and battery-powered multibillion dollar VR products are currently available to the anesthesiology community for a few hundred dollars per unit (e.g., the Meta Quest2 helmet). More widespread clinical use of VR distraction and pre-surgery patient education is recommended.

## Data Availability

The data supporting the conclusions of this article are publicly available.
